# Generation of special autosomal dominant polycystic kidney disease iPSCs with the capability of functional kidney-like cell differentiation

**DOI:** 10.1186/s13287-017-0645-8

**Published:** 2017-09-19

**Authors:** Jiahui Huang, Shumin Zhou, Xin Niu, Bin Hu, Qing Li, Feng Zhang, Xue Zhang, Xiujuan Cai, Yuanlei Lou, Fen Liu, Chenming Xu, Yang Wang

**Affiliations:** 1 Institute of Microsurgery on Extremities, Shanghai Jiao Tong University Affiliated Sixth People’s Hospital, Shanghai, People’s Republic of China; 2 Institute of Urology First Affiliated Hospital of Nanchang University, Nanchang, People’s Republic of China; 3 Department of Clinical Laboratory, Sixth Affiliated Hospital of Sun Yat-Sen University, Guangzhou, People’s Republic of China; 4 State Key Laboratory of Genetic Engineering, School of Life Sciences, Fudan University, Shanghai, People’s Republic of China; 5 Key Laboratory of Regenerative Biology, South China Institute for Stem Cell Biology and Regenerative Medicine, Guangzhou Institutes of Biomedicine and Health, Chinese Academy of Sciences, Guangzhou, People’s Republic of China; 6 Institute of Embryo-Fetal Original Adult Disease Affiliated to Shanghai Jiao Tong, University School of Medicine, Shanghai, People’s Republic of China

**Keywords:** Induced pluripotent stem cells, Autosomal-dominant polycystic kidney disease, Differentiation, Kidney cells, *SAMSN1*

## Abstract

**Background:**

Human induced pluripotent stem cells (iPSCs) have been verified as a powerful cell model for the study of pathogenesis in hereditary disease. Autosomal dominant polycystic kidney disease (ADPKD) is caused by mutations of *PKD* or non-*PKD* genes. The pathogenesis of ADPKD remains unexplored because of the lack of a true human cell model.

**Methods:**

Six ADPKD patients and four healthy individuals were recruited as donors of somatic cells from a Chinese ADPKD family without mutations of the *PKD* genes but carrying *SAMSN1* gene deletion. The ADPKD-iPSCs were generated from somatic cells and were induced into kidney-like cells (KLCs) by a novel three-step method involving cytokines and renal epithelium growth medium. Furthermore, we analyzed functional properties of these KLCs by water transportation and albumin absorption assays.

**Results:**

We successfully generated iPSCs from ADPKD patients and differentiated them into KLCs that showed morphological and functional characteristics of human kidney cells. Further, we also found that ADPKD-iPSC-KLCs had a significantly higher rate of apoptosis and a significantly lower capacity for water transportation and albumin absorption compared to healthy sibling-derived differentiated KLCs. Furthermore, knockdown of *SAMSN1* in control iPSCs may attenuate differentiation and/or function of KLCs.

**Conclusions:**

These data show that we have created the first iPSCs established from ADPKD patients without mutations in the *PKD* genes, and suggest that the deletion mutation of *SAMSN1* might be involved in the differentiation and/or function of KLCs. ADPKD-iPSC-KLCs can be used as a versatile model system for the study of kidney disease.

**Electronic supplementary material:**

The online version of this article (doi:10.1186/s13287-017-0645-8) contains supplementary material, which is available to authorized users.

## Background

Induced pluripotent stem cells (iPSCs), which were first reported by Yamanaka in 2006, are usually generated by reprogramming somatic cells by introducing a number of pluripotent factors, generally *OCT4*, *SOX2*, *KLF4*, *C-MYC*, *LIN28* and *NANOG* [1–3]. iPSCs are characterized by an unlimited proliferative capacity and can be differentiated into the majority of cell types both in vivo and in vitro, offering an ideal tool for studying molecular and cellular mechanisms of hereditary diseases in vitro [4–7]. Autosomal dominant polycystic kidney disease (ADPKD) is a common life-threatening inherited renal disorder, characterized by the progressive formation of renal cysts and various extra-renal manifestations such as intracranial arterial aneurysms, and has a prevalence of approximately 1 in 400–1 in 1000 live births [8–11]. ADPKD results in severe destruction of normal renal parenchyma and eventually leads to renal failure. The majority of ADPKD patients ultimately enter end-stage renal disease (ESRD) in their 50s and 60s, and have to undergo dialysis therapy for the rest of their lives or receive kidney transplantation [12]. Genetic defects in two genes named *PKD1* (*polycystin-1*; *PC1*) or *PKD2* (*polycystin-2*; *PC2*) are associated with ADPKD. Mutations of these two *PKD* genes account for approximately 91% of the pathogenesis of the disease [13–15]. However, in approximately 9% of ADPKD cases mutations have not been detected [15–17]. In the absence of credible human cell models, the pathogenesis of ADPKD has not been investigated thoroughly. The construction of a cell model of ADPKD in vitro is an urgent task and is the key to discovering the pathogenesis of ADPKD.

In this study, we demonstrated the generation and characterization of iPSCs from ADPKD patients without *PKD1/PKD2* mutations. These iPSCs are indistinguishable from human embryonic stem cells (hESCs) with respect to colony morphology, passaging, surface and pluripotent markers, normal karyotype, DNA methylation, and differentiation potential. We also describe and illustrate the efficient **directed differentiation of ADPKD-iPSCs into functional kidney-like cells (KLCs)** in vitro; in addition, we reveal that low-level expression of the *SAMSN1* gene can attenuate differentiation and function of KLCs in ADPKD. We are the first to establish iPSCs from ADPKD patients without mutations in the *PKD1* or *PKD2* genes, and our results show that a deletion mutation in the *SAMSN1* gene might be involved in the differentiation and/or function of KLCs in ADPKD-iPSCs.

## Methods

### Cell culture

**Fig. 1:**
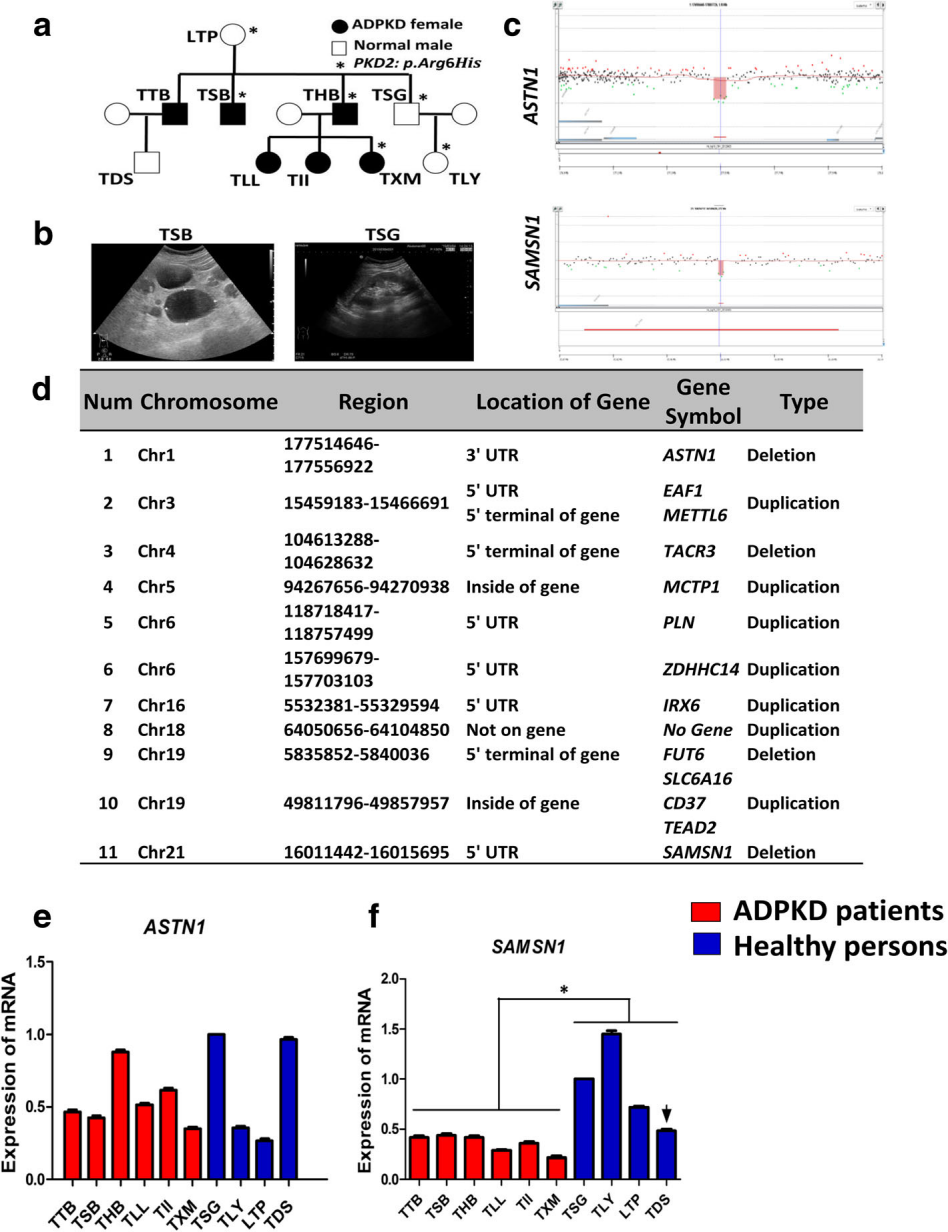
Genotyping of the special ADPKD family in this study. (**a**) Family pedigree. *Members with a missense mutation c.17G > A, *p. Arg6His* in *PKD2*. (**b**) Diagnostic ultrasonogram of the representative affected person (TSB) and normal person (TSG). (**c**) Upstream deletions of the *ASTN1* and *SAMSN1* genes identified in patient TSB by comparative genomic hybridization microarray technology. (**d**) List of all 11 CNVs in the genomes of TSB cells compared to TSG cells. (**e**) qPCR analysis of the *ASTN1* gene expression in ADPKD patients and healthy persons. Data presented as mean ± standard deviation from three independent sets of experiments. (**f**) qPCR analysis of *SAMSN1* expression in ADPKD patients and healthy persons. Data presented as mean ± standard deviation from three independent sets of experiments. *P < 0.05. ADPKD autosomal dominant polycystic kidney disease, TTB, TSB, THB, TLL, TII, TXM, TSG, TLY, LTP, TDS names of family members (Color figure online)

As shown in Fig. 1a, a Chinese ADPKD family containing ten living persons was selected for this study. The mother (LTP) and one of her sons (TSG) exhibited healthy phenotypes whereas the other three sons (TSB, TTB and THB) showed characteristic phenotypes of ADPKD. TSB exhibited the most severe, bilateral renal cysts of various sizes while THB had a relatively mild renal cyst phenomenon which was verified by ultrasound diagnosis (Fig. 1b). However, three daughters of THB (TLL, TII and TXM) exhibited ADPKD phenotypes while the other two grandchildren (TDS and TLY) of LTP exhibited healthy phenotypes. After ultrasound diagnosis, six members (TTB, TSB, THB, TLL, TII and TXM) exhibited a renal cyst phenomenon and were defined as “ADPKD patients”, while the others (TSG, TLY, LTP and TDS) exhibited healthy phenotypes and were defined as “healthy persons”. Human fibroblast cells (HFCs) and blood samples were obtained after the individuals provided informed consent; the consent forms are available upon request from the ethics committee of the First Affiliated Hospital of Nanchang University who approved the procedure. HFCs were maintained in fibroblast medium with Modified Eagle’s medium/Nutrient Mixture F12 (DMEM/F12; Invitrogen, Rockville, MD, USA) supplemented with 10% fetal bovine serum (FBS; Hyclone, Logan, UT, USA) at 37 °C. Blood samples were well preserved in liquid nitrogen.

### Gene mutation analysis

#### Sanger sequencing analysis

A long-range PCR (LR-PCR) strategy followed by nested PCR was used for mutational analysis of the *PKD1* gene. The duplicated region of *PKD1* was amplified in eight specific long fragments by LR-PCR (exons 1, 2–7, 8–12, 13–15, 15–21, 22, 23–28 and 29–34) [18] using TaKaRa LA Taq™ (TaKaRa Bio Inc.). Exons 1–34 of *PKD1* were then amplified by nested PCR using these LR templates and exons 35–46 were directly PCR amplified and sequenced in both directions. The proband without pathogenic mutation in the *PKD1* gene was subsequently analyzed by mutational screening of the *PKD2* gene via Sanger sequencing. Exons 1–15 of *PKD2* including the adjacent 30–50 bp intronic sequence were amplified from genomic DNA according to a method reported previously with little modification [19]. PCR amplification primers for the various LR-PCR fragments are presented in Table 1.

**Table 1:** Primers for *PKD1* and *PKD2*

Fragment	Size (kb)	Exons	Forward primer	Reverse primer	Temperature (°C)
*PKD1*					
L1	2.2	1	CCATCCACCTGCTGTGTGACCTGGTAAAT	CCACCTCATCGCCCCTTCCTAAGCAT	68
L2	4.6	2–7	ATTTTTTGAGATGGAGCTTCACTCTTGCAGG	CGCTCGGCAGGCCCCTAACC	68
L3	4.2	8–12	CCGCCCCCAGGAGCCTAGACG	CATCCTGTTCATCCGCTCCACGGTTAC	68
L4	4.4	13–15	TGGAGGGAGGGACGCCAATC	GTCAACGTGGGCCTCCAAGT	68
L5	3.4	15–21	AGCGCAACTACTTGGAGGCCC	GCAGGGTGAGCAGGTGGGGCCATCCTA	70
L6	0.3	22	GAGGCTGTGGGGGTCCAGTCAAGTGG	AGGGAGGCAGAGGAAAGGGCCGAAC	64
L7	4.2	23–28	CCCCGTCCTCCCCGTCCTTTTGTC	AAGCGCAAAAGGGCTGCGTCG	68
L8	5.8	29–34	GGCCCTCCCTGCCTTCTAGGCG	GTTGCAGCCAAGCCCATGTTA	68
35–37	0.7	35–37	GGGATGAATTCACAGCCTAC	GGAGACAAGAGACGGAGGT	62
38–40	1.1	38–40	AAGCCCTGCTGTCACTGT	TACTCCCTTGTCCTTGGC	56
41–43	1.1	41–43	GGGAGTAGTTCTCCAGGAGTG	CGAGAAATCTGTCTGCTTGC	62
44–46	1.1	44–46	GGCTGCAAGCAGACAGATT	GCGGTGTCCACTCCGACTCC	56
*PKD2*					
1-1	0.5	1	AGAGGGAGGCGGGCCAAAGG	CGGGCGCCACTCTACGTCCA	62
1-2	0.4	1	GTGGAGCCGCGATAACCCCG	AGGCGGAACGCAGAGGGGAT	62
2	0.5	2	TTGTGCTTTATTTTCCCTTTTGCCA	TGCCTCTCCCGTCCTGTGTT	59
3–4	2.5	3–4	AGGGGAAAGGAAGGCAAGGGTGA	TGCCTTGGTGAAGGTGTCAGGGA	65
5–6	4.0	5–6	GCCAGGTCAGGCACAGTACCC	AGCGTGGCTGAGAGCATACTGT	63
7–8	4.5	7–8	TGGCAGGGCTTAACACTTTCCATTT	TCTTGAGAAGCAGTGACAACTCTGA	65
9–10	4.7	9–10	ACCGTGCCCAGCTTGTGTTT	CTGCCGTGGAAGGTCAAGGG	65
11–13	2.9	11–13	CCAGCACGTACTTGTTGAATGGCC	GGGAACTGCCTGGTCTCATGTGG	65
14–15	1.0	14–15	GCCAGTGGGGCTGAAAAGACA	AGCATCCTATGGTGGTCAGGGCA	70

The pathogenicity of novel missense variants was evaluated computationally by SIFT, PolyPhen2 and AlignGVGD prediction programs through analyzing interspecies sequence variations. When all programs classified it as damaging and no other definite mutation was found in the same patient, the variant was considered likely to be pathogenic. The variant would be regarded as a polymorphism in cases where all scores denied its pathogenicity. Otherwise, they were classified as “likely polymorphisms”. Furthermore, pedigree cosegregation analysis of the potential pathogenic mutations in *PKD2* was performed in all available members.

#### Comparative genomic hybridization microarray

Genomic DNA extracted from patient TSB together with sex-matched control DNA was fragmented by *Alu*I and *Rsa*I enzyme digestion. DNA labeling was conducted using an Agilent SureTag DNA Labeling Kit. Different fluorescence dyes were used for DNA labeling of patient DNA (Cy5-dUTP) and control DNA (Cy3-dUTP). The labeled products were mixed and hybridized onto Agilent SurePrint G3 human 1 × v1M microarray for 40 hours at 65 °C. DNA processing, microarray handling and scanning were conducted following the Agilent oligonucleotide comparative genomic hybridization (CGH) protocol (version 6.0). The microarray scanning profiles were processed by Agilent Feature Extraction 10.7.3.1. The extracted data were analyzed and plotted by Agilent Workbench 7.0. ADM-2 was selected as the statistical algorithm with the threshold set at 6.0 and Fuzzy Zero turned on.

#### Generation of iPSCs

The reprogramming process was developed as depicted diagrammatically in Additional file 1: Figure S1a. The key principle of the reprogramming approach is that the expression of four individual transcription factors (*OCT4*, *SOX2*, *KLF4* and *c-MYC*) and the expression of a cocktail of transcription factors (*OCT4*, *SOX2*, *KLF4* and *c-MYC*) erases the active epigenetic network of a somatic cell and reopens a “ground state” of undifferentiated pluripotency [1, 2]. We introduced the retroviruses containing human *OCT4*, *SOX2*, *KLF4* and *c-MYC* produced in 293 T cells (PLAT-A packaging cells [20]) into HFCs (retroviral vectors obtained from Dr Duanqing Pei’s laboratory, Guangzhou, China). HFCs from TSB, THB and TSG were used to reprogram into iPSCs. Approximately 4 × 10^4^ HFCs were cultured until they reached 80% confluence and were transduced in fibroblast medium with a cocktail of retroviruses as reported previously [21, 22]. At day 2 post infection, medium was changed to DMEM/F12 containing 20% FBS and 50 μg/ml Vitamin C (Vc) (sodium l-ascorbate; Sigma, St Louis, MO, USA). At day 6, infected HFCs were trypsinized and reseeded onto feeder cells (mitotically inactivated murine embryonic fibroblasts) and cultured in DMEM/F12 medium supplemented with 10% FBS, 50 μg/ml Vc, 1 mM/ml valproic acid (VPA; Merck, Darmstadt, Germany), 1 mM glutamax (Invitrogen), 0.1 mM nonessential amino acids (NEAA; Invitrogen), 0.1 mM β-mercaptoethanol (β-ME; Invitrogen) and 8 ng/ml basic fibroblast growth factor (bFGF; Shenzhen Symmix Industry, Shenzhen, China). VPA was added on days 7–22. Effectiveness of the cell transduction was assessed by the appearance of GFP-positive cells under fluorescent microscopy. Human ESC-like colonies appeared at around day 20 post infection and were picked up manually at around day 25. Picked iPSCs were routinely maintained on Matrigel® (Becton Dickinson, Franklin Lakes, NJ, USA) in mTeSR1 medium (Stemcell Technologies, Madison, WI, USA).

#### Alkaline phosphatase staining

The iPSCs were fixed with 4% paraformaldehyde (PFA) for 2 minutes at room temperature (RT) followed by two washes with sterile phosphate buffered saline (PBS), and then rinsed with Tris-buffered saline containing Tween-20 (TBST; 20 mM Tris–HCl, pH 7.4; 0.15 M NaCl, 0.05% Tween-20). Freshly prepared alkaline phosphatase (AP) staining solution (Sidansai, Shanghai, China) was added to the colonies and they were incubated for 15 minutes at RT in the dark. The staining solution was then aspirated and the colonies were washed twice with sterile PBS and analyzed by light microscopy.

#### Immunofluorescence staining

The iPSCs were fixed in 4% PFA for 20–30 minutes at RT, washed twice with sterile PBS, permeabilized in 0.5% Triton X-100 for 20 minutes and blocked with 5% bovine serum albumin (BSA) solution in sterile PBS for 2 hours. The cells were then incubated with primary antibody in 0.5% BSA solution at 4 °C overnight. Next day, the primary antibody solution was aspirated, cells were washed five times for 30 minutes, and then incubated with the corresponding secondary antibody solution for 1 hour at 37 °C. Cells were washed, stained with 4,6-diamino-2-phenylindole (DAPI; Sigma) for 5 minutes at RT, washed and covered in sterile PBS, and finally photographed under a fluorescence microscope (Leica Microsystems, Wetzlar, Germany).

For confocal imaging of primary cilia, cells were grown on glass coverslips up to day 29. The cells were fixed and acetylated alpha-tubulin immunofluorescence staining was performed as described previously [23]. Cells were imaged on a Zeiss LSM 5 Pascal confocal microscope (Carl Zeiss, Oberkochen, Germany) using a 1.4 numerical aperture in plane or a 2.8 numerical aperture in stack scan mode. Images were deconvolved using Zeiss LSM Examiner software (version 4.0.0.241). All antibodies used are presented in Table 2.

**Table 2:** Primary antibodies

Antibody	Isotype	Dilution	Source
OCT4	Rabbit IgG	1:50	CST 2840 s
SSEA-4	Mouse IgG	1:50	Abcam 4755
TRA-1-60	Mouse IgM	1:100	CST 4746
TRA-1-81	Mouse IgM	1:50	CST 4745
SOX2	Mouse IgG	1:100	CST 2748 s
AFP	Mouse IgG	1:100	CST 3903 s
Nestin	Rabbit IgG	1:200	Abcam ab105389
Bry	Rabbit IgG	1:200	Abcam ab20680
Desmin	Rabbit IgG	1:100	CST 5332 s
Pax2	Rabbit IgG	1:100	Abcam ab79389
AQP1	Rabbit IgG	1:100	CST Sc52623
βIII-tubulin	Mouse IgG	1:200	CST 4466 s
E-cad	Rabbit IgG	1:20	CST 3195
Synaptopodin	Mouse IgG	1:100	PROGEN Biotechnik SC6260
Anti-acetylated alpha tubulin	Mouse IgG	1:100	Abcam ab24610

#### In-vitro and in-vivo differentiation

To test the differentiation capacity of iPSC lines, iPSC colonies growing on Matrigel® were loosely detached by dispase treatment for 5 minutes, washed four times with DMEM/F12, scraped up with a glass pipette, and resuspended in DMEM/F12 medium containing 20% knockout serum replacement (KSR; Gibco, Thermo Fisher Scientific, Waltham, MA, USA), 1 mM glutamax, 0.1 mM NEAA and 0.1 mM β-ME. Embryoid bodies (EBs) were maintained on 1% agar-coated low-attachment plates and replenished every 2 days with fresh EB medium (DMEM/F12 containing 20% FBS). EBs were placed on Matrigel®-coated plates after 8 days in suspension, and then allowed to differentiate for another 18 days in EB medium before processing for immunofluorescence analysis. As for teratoma formation, iPSCs were washed with DMEM/F12, treated with dispase for 5 minutes at 37 °C, scraped up using a glass pipette, collected by centrifugation and resuspended in DMEM/F12 containing Matrigel®. Approximately 2 × 10^6^ iPSCs were injected into immune-compromised NOD-SCID mice (Weitonglihua, Beijing, China). Eight weeks after injection, teratomas were dissected, rinsed once with sterile PBS, fixed with 10% formalin, embedded in paraffin and cut into sections 4–5 μm thick. Hematoxylin/eosin staining was performed as reported previously [24].

#### Karyotype analyses

The iPSCs were cultured in six-well plates until they reached 80–90% confluence before mitotic arrest was induced by treatment with 20 μg/ml colcemid for 2 hours. Following incubation, the colonies were digested using 0.25% trypsin ethylene diamine tetraacetic acid (EDTA) (Invitrogen), and cells were centrifuged at 2000 × *g* for 5 minutes, resuspended in 7 ml of 0.075 M KCl, and incubated for 20 minutes at 37 °C. Prefixative solution composed of one part acetic acid and three parts methanol was added, mixed gently, and incubated for 40 minutes at 37 °C. After further centrifugation, the supernatant was removed. Cells were dropped onto a cold slide and incubated at 75 °C for 3 hours. Giemsa banding was performed following a standard protocol with incubations in 0.05% trypsin for 8 seconds and Giemsa staining dilution for 10 minutes. Imaging and karyotyping were performed using Meta Systems Band View software.

#### DNA fingerprinting using short tandem repeat analysis

To confirm whether iPSCs were derived from ADPKD fibroblasts, short tandem repeat (STR) analysis was performed by the DNA Sequencing Core Facility at Technology Biological Co., Ltd, Shanghai Boyi.

#### Flow cytometry analyses

The iPSCs were suspended with 0.05% trypsin for 10 minutes, centrifuged, and then resuspended in sterile PBS. The single cell suspension was fixed in 1% PFA for 15 minutes at 37 °C and permeabilized in 90% precooled methanol for 30 minutes. Both primary and secondary antibody incubations were carried out according to the manufacturer’s instructions. Control samples were stained with isotype-matched control antibodies. After washing, the cells were resuspended, filtered, and then used for flow cytometry (BD FACS Aria; Becton Dickinson). The antibodies used for flow cytometry are presented in Table 2.

#### Quantitative reverse transcription-polymerase chain reaction

RNA was extracted using Trizol reagent (Invitrogen). qPCR was performed using an ABI7900 Thermal Cycler Dice™ Real Time System (ABI, Foster City, CA, USA) and SYBR Green Premix EX Taq™ (Takara, Shiga, Japan). *GAPDH* was used for normalization and all items were measured in triplicate. Quantitative reverse transcription-polymerase chain reaction (RT-qPCR) and semi-quantitative PCR primers are summarized in Table 3.

**Table 3:** Primers for characterization

Gene	Forward sequence	Reverse sequence
*ACTB*	CCCAGAGCAAGAGAGG	GTCCAGACGCAGGATG
*endo-OCT4*	CCTCACTTCACTGCACTGTA	CAGGTTTTCTTTCCCTAGCT
*endo-Sox2*	CCCAGCAGACTTCACATGT	CCTCCCATTTCCCTCGTTTT
*Nanog*	TGAACCTCAGCTACAAACAG	TGGTGGTAGGAAGAGTAAAG
*Rex1*	TCGCTGAGCTGAAACAAATG	CCCTTCTTGAAGGTTTACAC
*AFP*	ATTGGCAAAGCGAAGCTG	GCTGTGGCTGCCATTTTT
*CK18*	AGCTCA ACGGGATCCTGCTGCACCTTG	CACTATCCGGCGGGGGTGGCTTTTG
*MSX1*	CACTATCCGGCGGGGGTGGCTTTTG	CGAGAGGACCCCGTGGATGCAGAG
*TBX1*	AGCGAGAAATATGCCGAGG	TTCGCGAAGGGATTGCT
*PAX6*	TTCGCGAAGGGATTGCT	TGCCCGTTCAACATCCTT
*SOX1*	TTTCCCCTCGCTTTCTCA	TGCAGGCTGAATTCGGTT
*PAX2*	AGATTCCCAGAGTGGTGTG	GGGTATGTCTGTGTGCCTGA

#### Methylation analysis of gene promoters

Bisulfite treatment was performed using a cytosine guanine dinucleotide (CpG) modification kit (Promega, Madison, WI, USA) according to the manufacturer’s recommendations. Amplified products were cloned into PCR2.1-TOPO (Takara). Twelve randomly selected clones were sequenced with the M13 forward and M13 reverse primers for each gene. PCR primers are presented in Tables 3 and 4.

**Table 4:** Primers for kidney-like cell differentiation

Gene	Forward sequence	Reverse sequence
*BRY*	GACTGCTTATCAGAACGAGG	TGTCAGAATAGGATTGGGAG
*PAX2*	AACGACAGAACCCGACTATG	ATCCCACTGGGTCATTGGAG
*AQP1*	ATTAACCCTGCTCGGTCCTT	ACCCTGGAGTTGATGTCGTC
*E-cad*	TCCCATGCCTACCTCACCTT	ACCCTGGAGTTGATGTCGTC
*Synaptopodin*	AGCCCAAGGTGACCCCGAAT	CCCTGTCACGAGGTGCTGGC
*WT1*	GGACAGAAGGGCAGAGCAACCA	GTCTCAGATGCCGACCGTACAA
*GADPH*	GTCTCCTCTGACTTCAACAGCG	ACCACCCTGTTGCTGTAGCCAA
*Lim1*	TCATGCAGGTGAAGCAGTTC	TCCAGGGAAGGCAAACTCTA

#### In-vitro kidney lineage differentiation studies

The kidney differentiation capacity of human ADPKD-iPSCs was developed as depicted diagrammatically in Fig. 4a. To induce differentiation of KLCs from ADPKD-iPSC colonies, colonies of H9 ESCs and ADPKD-iPSCs were cut into uniform-sized pieces, transferred into six-well plates precoated with Matrigel® for 1 hour, and cultured initially in mTeSR1 medium until 30% confluent. To induce differentiation, mTeSR1 medium was replaced with stage 1 medium with DMEM/F12, containing 1% Glutamax, 1% NEAA and 10% FBS, and supplemented with bone morphogenic protein 7 (BMP7), human vascular endothelial growth factor (hVEGF), bFGF and Activin-A (all from HumanZyme, Chicago, IL, USA) during days 1–3 only (all at 10 ng/ml) and with 5 μM lithium for 14 days. To initiate differentiation toward the intermediate mesoderm (IM), cells were cultured for another 7 days with retinoic acid (RA; HumanZyme) supplemented with stage 1 medium without Activin-A. To induce renal precursor or mature cell differentiation, the cells were cultured with stage 1 medium in various combinations with renal epithelium growth medium (REGM; BioWhittaker, Walkersville, MD, USA) for another 7 days. The medium was changed every 2 days.

#### Proliferation assay studies

In-vitro cell proliferation assays were evaluated using a cell counting kit 8 (CCK8; Dojindo Molecular Technologies, Kumamoto, Japan) according to the manufacturer’s instructions. The cells differentiated at days 21–28 were used for proliferation assays by CCK8 reagent. Briefly, 720 μl of fresh medium and 80 μl of the CCK8 solution were added to each well, and cells were incubated at 37 °C for 1 hour. A blank well contained only the CCK8 reagent and medium without any cells. The absorbance at 450 nm was measured using an automatic microplate reader (BioTek Instruments, Winooski, VT, USA). All experiments were performed in triplicate.

#### Apoptosis studies

For apoptosis analysis, both apoptotic and necrotic cells in kidney differentiation cultures were measured using the Annexin V FITC/propidium iodide (PI) apoptosis detection kit (Dojindo) following the manufacturer’s protocol at 21–25 days. In brief, both adherent and floating cells were collected, 10^6^ cells were washed twice with cold sterile PBS and then resuspended in Annexin V-FITC binding buffer. FITC-conjugated Annexin V (100 μl/sample) was added and cells were incubated for 10 minutes at RT in the dark. Cells were then centrifuged and resuspended in binding buffer, and PI was added (100 μl/sample). Samples were kept on ice and incubated for 20 minutes in the dark. The total percentage of apoptotic cells was measured by counting the number of FITC^+^ and FITC^+^/PI^+^ stained cells by Guava easyCyte6HT™ flow cytometry (5000 events/gate). Representative data from one of three independent experiments were analyzed using its built-in INCYTE (version 2.7) software (EMD Millipore, Merck).

#### Water transport assay

Cells cultured in differentiation medium for 28 days and the human kidney (HK2) positive cells were rinsed with PBS and loaded with CFSE (Invitrogen) for 10 minutes. After washing with sterile PBS, cells were incubated in a hypotonic solution (0.06% NaCl in water). The fluorescence intensity in the supernatant was then measured by Synergy™ HT (Bio-Tek).

#### Cell permeability

A cell permeability assay was used to determine the endocytic uptake of fluorescence-labeled albumin as further evidence of glomerulus-like or podocyte-like functional characteristics. The protocol was as reported previously [25]. iPSCs differentiated for 28 days and primary podocyte-positive cells were cultured in serum-free medium with/without rhodamine-labeled albumin (0.5 mg/ml; Abcam, Cambridge, MA, USA) at 37 °C for 1 hour, while control cells were cultured at 4 °C. After washing with sterile PBS, the cells were fixed in 4% PFA and counterstained with DAPI solution. Finally the cells were photographed under a fluorescence microscope (Leica microscopy) and the fluorescence intensity was measured by Image J 1.48.

#### Knock down of the *SAMSN1* gene

The short hairpin RNAs (shRNAs) presented in Table 5 were used to target *SAMSN1*. Sh*SAMSN1* and negative control shRNA were synthesized and inserted into a lentivirus shuttle vector containing an enhanced green fluorescent protein (*EGFP*) reporter gene and a puromycin antibiotic resistance gene. Expression of the shRNA was driven by the H1 promoter. Recombinant lentiviruses expressing *SAMSN1-shRNA* or negative control shRNA (*Lv-shSAMSN1* and Lv-shNC) were produced in 293 T cells. TSG-iPSCs were infected with concentrated *Lv-shSAMSN1* or Lv-shNC virus in serum-free medium. The supernatant was replaced with complete culture medium after 6 hours. The stable knockdown cells were established by selection in complete culture medium containing puromycin (1.0 μg/ml) for approximately 14 days and then validated by qRT-PCR analysis. All of the primers are presented in Table 5.

**Table 5:** Primers for *SAMSN1* knockdown

Name	Sequence (5′ → 3′)
shRNA-391	GGAGAGAATGCCCACCCATAT
shRNA-601	GCCAGAGTGCATACGGATTTC
shRNA-707	GGACAGGAATGTTGAACAATA
shRNA-840	GGAGTTCCTAGAGAGGATTCA
shNC	GTTCTCCGAACGTGTCACGT

### Statistical analysis

All assays were performed in triplicate. Results are reported as the mean ± SD, and statistical significance was displayed as *P* < 0.05 and *P* < 0.01. Significant differences between two groups were determined by the independent Student’s *t* test.

## Results

### Genotyping of the special ADPKD family

We first collected blood samples from all ten members of this family, including TSB (patient) and TSG (healthy sibling). Then we sequenced the *PKD1* and *PKD2* genes in patient TSB and his healthy brother TSG by Sanger sequencing. The proband (patient TSB) without pathogenic mutation in the *PKD1* gene was subsequently analyzed by mutational screening of the *PKD2* gene via Sanger sequencing. A novel missense mutation c.17G > A, *p. Arg*6*His* in *PKD2* was found in the proband. As shown in Fig. 1a, however, this mutation was also found in his healthy relatives (LTP, TSG and TLY), and was found to be absent from other affected family members (TTB, TLL and TII); thus the variant did not segregate with the disease in the family. According to our prediction standard, the mutation in *PKD2* c.17G > A, *p. Arg*6*His* was therefore predicted to be a polymorphism (Additional file 2: Figure S2).

We then hypothesized that other changes in the genome might play a key role in this special ADPKD family. TSB and TSG were analyzed by CGH microarrays. Eleven genes’ copy number variation (CNV) regions were detected (Fig. 1d), including deletions of *ASTN1* and *SAMSN1* mutations (Fig. 1c). However, no apparent deletion or duplication mutations were found at the loci of *PKD1* and *PKD2* genes (Additional file 3: Figure S3a). We then validated all CNV regions using RT-qPCR. Nine of eleven did not pass the verification and CNVs containing the 5′ upstream sequence of genes *ASTN1* and *SAMSN1* were consistent with the phenotypes of TSB and TSG (Additional file 3: Figure S3b). We further confirmed these two CNVs by RT-qPCR in all of the family members (Fig. 1e, f). Only CNV containing the 5′ upstream sequence of gene *SAMSN1* corresponded to the phenotype of individuals in this family (Fig. 1f) with the exception of TDS (Fig. 1f, arrowhead). So far, TDS did not exhibit renal cyst phenomenon by ultrasound diagnosis, while the *SAMSN1* gene expression in TDS displayed a relatively low level like ADPKD patients. Because the ADPKD patients usually take a long time to become symptomatic and TDS is only a 15-year-old boy, we inferred that TDS might exhibit a renal cyst phenomenon at an older age.

We can conclude from these two experiments that *PKD* mutations can be ruled out in this ADPKD family. However, CNV of the *SAMSN1* gene was identified as a possible candidate and consequently we would then proceed to validation analysis.

### Characterization of ADPKD-iPSCs

**Fig. 2:**
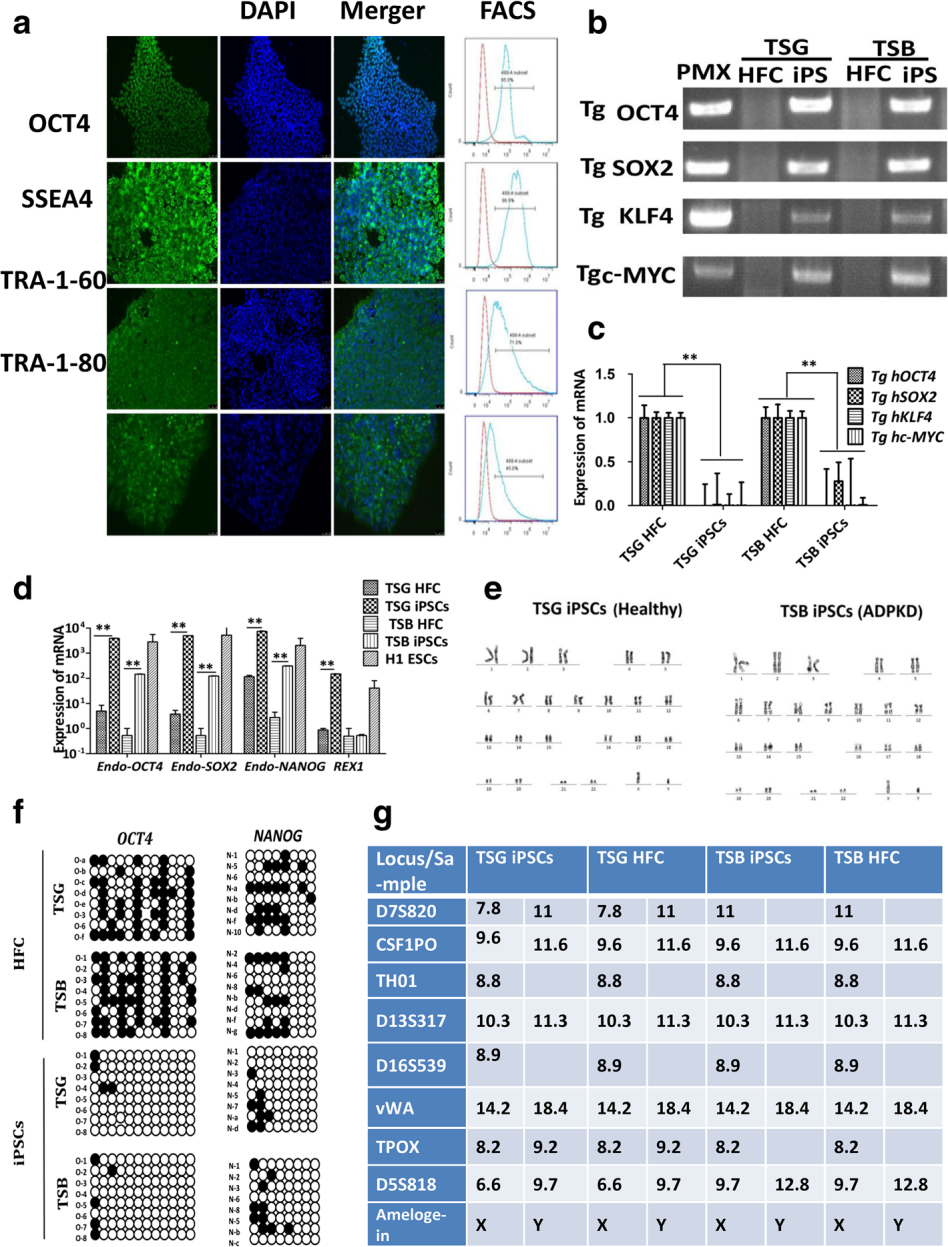
Generation and characterization of ADPKD-iPSCs. (**a**) Immunofluorescence staining and FCM analysis of ADPKD-iPSC colonies. Expression of iPSC specific proteins (OCT4, SSEA4, TRA-1-60 and TRA-1-81) (first column) with corresponding DAPI-stained nuclei (second column) and merged images (third column). These cells were also analyzed by FCM and positive rates were tested. Bar = 50 μm. (**b**) Semi-quantitative PCR results showing that expressions of exogenous genes were overregulated in iPSCs after day 6 during programming. (**c**) qPCR showing that expressions of exogenous genes in iPSCs were silent after day 19 during programming. Data presented as mean ± standard deviation from three independent sets of experiments. **P < 0.01. (**d**) qPCR results showing upregulated expression of endogenous iPSC specific genes in healthy or ADPKD-iPSCs. Human embryonic stem cells (H1 ESCs) acted as a positive control. Data presented as mean ± standard deviation from three independent sets of experiments. **P < 0.01. (**e**) ADPKD specific iPSC colonies showing a normal 46XY karyotype. (**f**) Methylation status of eight CpGs analyzed (one per row) in the promoter region of both *OCT4* and *NANOG* genes from twelve or eight randomly sequenced clones represented as 8 × 12 and 8 × 8 matrices, respectively, for both iPSCs and human fibroblast cells (HFCs). Open circles indicate the unmethylated state and dark, filled circles indicate the methylated state, which overall indicated that the loci tested are highly methylated in HFCs, while they have been reprogrammed to the unmethylated state in the iPSC colonies. (**g**) Genomic fingerprint analysis of TSG and TSB in both iPSCs and their corresponding HFCs. ADPKD autosomal dominant polycystic kidney disease, ESC embryonic stem cell, iPSC induced pluripotent stem cell, TSB, TSG names of family members (Color figure online)

Six iPSC clones from these individuals (TSB, THB and TSG) were generated in our study, and three iPSC clones (TSG iPSCs, THB iPSCs and TSB iPSCs) were selected for further characterization. To detect the expression of pluripotency markers in the picked colonies, iPSCs that were positive for AP activity (Additional file 1: Figure S1b) were analyzed by immunostaining and flow cytometry (FCM) for OCT4, SSEA4, TRA-1-60 and TRA-1-81, and the results showed that all four pluripotency marker proteins were upregulated (Fig. 2a). To further confirm that the selected colonies were really iPSCs, exogenous and endogenous pluripotency marker genes were analyzed by semi-quantitative PCR and qPCR in the genomic DNA of iPSCs. As shown in Fig. 2b, all integration exogenous pluripotency genes were overexpressed in all iPSCs compared to those in fibroblasts on day 6. Nineteen days later, the integration exogenous transcription factors were silenced in all iPSCs by host cells (Fig. 2c), whereas endogenous pluripotency factors were activated in all iPSCs (Fig. 2d), which proved that the selected colonies had been reprogrammed successfully. H1 ESCs were positive cells.

In order to analyze whether the genome of established iPSCs was stable and normal, karyotype analysis was performed as described previously [26]. As shown in Fig. 2e, both iPSC lines (TSG, TSB and THB) displayed a normal karyotype of 46XY (data for THB not shown). We then studied the methylation status of CpG in the promoter regions of key transcription factors by bisulfite sequencing, and found that the promoters of *OCT4* and *NANOG* displayed extensive DNA demethylation in iPSCs, in contrast to their parent fibroblasts (Fig. 2f). In addition, genetic STR analysis confirmed that the iPSCs were derived from this individual’s fibroblasts and were not contaminated with other cell lines grown in our laboratory (Fig. 2g). All of the characterization results of the three iPSC lines show a similar tendency.

### In-vitro and in-vivo differentiation of ADPKD-iPSCs

**Fig. 3:**
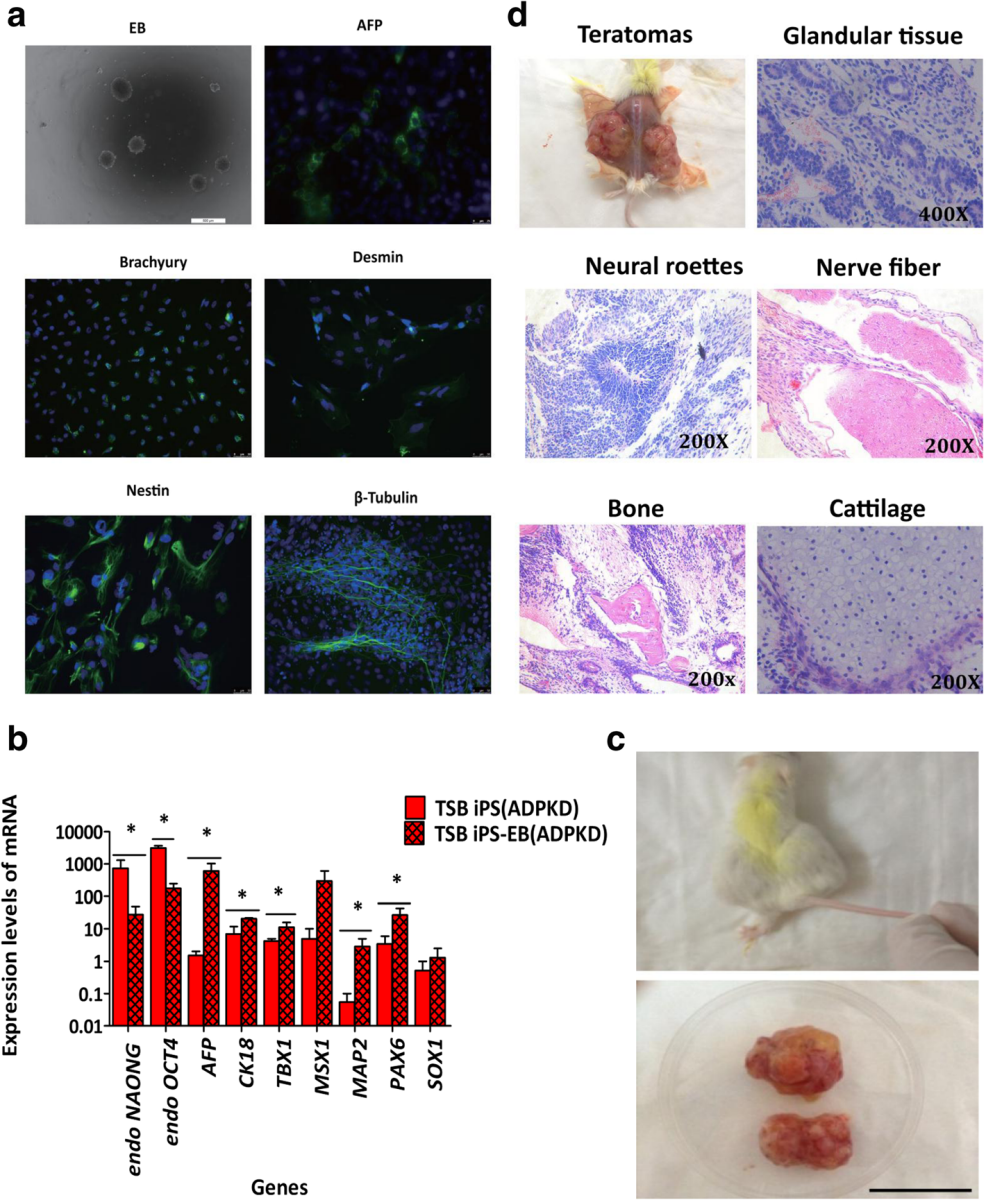
In-vitro and in-vivo differentiation of ADPKD-iPSCs. (**a**) Embryoid body (EB) formation by ADPKD-specific iPSCs in suspension culture. Differentiated EBs expressed markers from all three germ layers, including α-fetoprotein (AFP; endoderm, bar = 25 μm), Nestin and Desmin (mesoderm), Brachyury: BRY and β_III_-tubulin (ectoderm). Bar = 50 μm. (**b**) qPCR analysis showing differences in gene expression patterns between undifferentiated iPSCs and differentiated EBs. Undifferentiated iPSCs expressed high levels of endogenous *OCT4* and *NANOG* genes while EBs expressed high levels of marker genes of all three layers. Data presented as mean ± standard deviation from three independent sets of experiments. *P < 0.05. (**c**) Teratomas evident following the injection of undifferentiated ADPKD-specific iPSCs into immunodeficient mice. Bar = 1 cm. (**d**) Hematoxylin and eosin staining of tissues from all three germ layers. Bar = 1 cm. ADPKD autosomal dominant polycystic kidney disease, iPS induced pluripotent stem cell, TSB name of family member (Color figure online)

To further confirm the pluripotency of these special ADPKD-iPSCs, in-vitro and in-vivo differentiation capability were performed as described. For in-vitro EB differentiation, ADPKD-iPSC gobbets were maintained in suspension culture for EB formation for about 8 days followed by 18 days in attachment culture in order to differentiate into EBs (Fig. 3a, light view); protein markers of three germ layers were then analyzed by fluorescent immunostaining. As shown in Fig. 3a, the expression and localization of marker proteins representing endoderm cells (α-fetoprotein; AFP), mesoderm cells (Desmin, Brachyury: BRY), and ectoderm cells (β_III_-tubulin, Nestin) could be clearly detected, which demonstrated the pluripotency of ADPKD-iPSCs. Furthermore, qPCR analyses of more extensive marker genes of the three germ layers and pluripotency markers were also performed to confirm EB differentiation. As expected, the expressions of differentiation markers (*AFP*, *CK18*, *TBX1*, *MSX1*, *MAP1*, *SOX1* and *PAX6*) in EB differentiated cells were increased compared to that of in ADPKD-iPSCs while the expressions of pluripotency markers (*endo-NANOG* and *endo-OCT4*) were decreased (Fig. 3b).

In addition, the pluripotent properties of these iPSCs were assessed by teratoma formation in vivo. NOD-SCID mouse recipients were employed for differentiation by iPSCs injection. The formation of teratomas (Fig. 3c) was observed (3/3 mice), which produced derivatives of the three germ layers including rather complex structures in the case of teratomas (Fig. 3c). Taken together, our analyses of ADPKD-iPSCs derived from retrovirally transduced ADPKD patient fibroblasts confirmed their pluripotent potential.

### Directed differentiation of ADPKD-iPSCs into KLCs

**Fig. 4:**
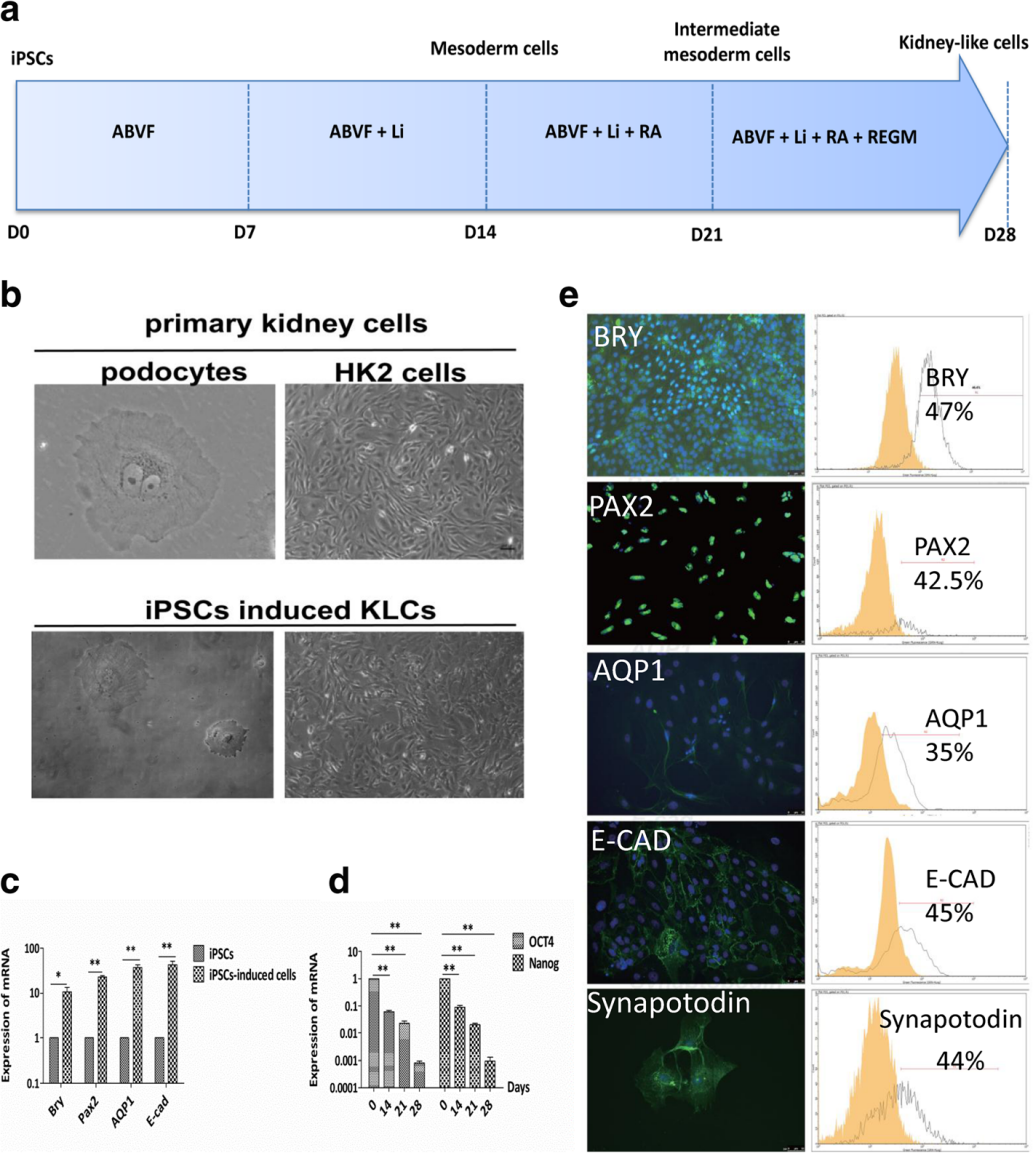
Direct differentiation of ADPKD-iPSCs into kidney-like cells (KLCs). (**a**) Scheme showing the stepwise protocol used for producing KLCs from ADPKD-iPSCs and the time needed. (**b**) Morphology of induced ADPKD-iPSCs is similar to podocytes and human kidney (HK2) cells. Bar = 100 μm. (**c**) Upregulation of marker genes of each stage during differentiation from iPSCs into functional KLCs. Values (mean of three replicates) are referred to the undifferentiated iPSCs. Data presented as mean ± standard deviation from three independent sets of experiments, *P < 0.05, **P < 0.01. (**b**) Pluripotency of iPSCs decreased during induction to KLCs. Data are averages and standard deviations of three independent experiments. Values (mean of three replicates) are referred to the undifferentiated iPSCs. **P < 0.01. (**e**) Immunofluorescence and FCM results of marker genes of each step of induction. BRY is a marker of mesoderm cells; PAX2 a marker for intermesoderm cells; and synaptopodin, AQP1, and E-cadherin (E-CAD) are markers for KLCs. Bar = 50 μm. iPSC induced pluripotent stem cell, RA retinoic acid, REGM renal epithelium growth medium, ABVF Activin-A, BMP7, hVEGF and bFGF

In order to model the progress of ADPKD in vitro, it is necessary to first induce iPSCs to differentiate into the kidney lineage cells. The stepwise differentiation method we set up simulated the process of kidney generation in embryo development through three main phases: mesoderm, intermediate mesoderm (IM) and KLCs. As depicted diagrammatically in Fig. 4a, iPSCs were induced in ABVF (Activin-A, BMP7, hVEGF and bFGF) condition medium for at least 28 days by adding lithium chloride, retinoic acid (RA) and REGM. At 28 days, examination of the differentiated ADPKD-iSPCs showed that they had developed into two morphologically different cell types. One was large, often multinucleated and arborized cells with cytoplasmic extensions (Fig. 4b, lower left). The morphology was comparable to conditionally immortalized human podocytes (Fig. 4b, upper left) [27]. The other type consisted of fusiform and fibroblast-like cells (Fig. 4b, lower right) which appeared similar to human kidney (HK2) cells under the light microscope (Fig. 4b, upper right). We also performed immunofluorescence staining of primary cilium, which is a surface feature of podocytes (Additional file 1: Figure S1c, red arrow), and found no difference in cilium formation between ADPKD-iPSCs and normal iPSCs.

To map the process of differentiation of ADPKD-iPSCs into KLCs, markers of genes corresponding to the three phases were analyzed by qPCR. The results showed that the expression of genes characteristic of mesoderm, IM and KLCs were increased (Fig. 4c) whereas those of the pluripotency genes (*OCT4* and *NANOG*) were decreased (Fig. 4d) during kidney lineage cell differentiation compared to iPSCs. Human fetal kidney cDNA was used as positive control. Furthermore, the proportions of BRY^+^ cells (47%), PAX*2* ^*+*^ cells (42.5%), AQP1^+^ cells (35%), E-cadherin^+^ cells (45%) and synaptopodin^+^ cells (44%) were obtained from FCM assay (Fig. 4e, right), and immunofluorescence staining also confirmed these results (Fig. 4e, left).

### Differentiated iPSCs from ADPKD patients and healthy individuals expressed different phenotypes

**Fig. 5:**
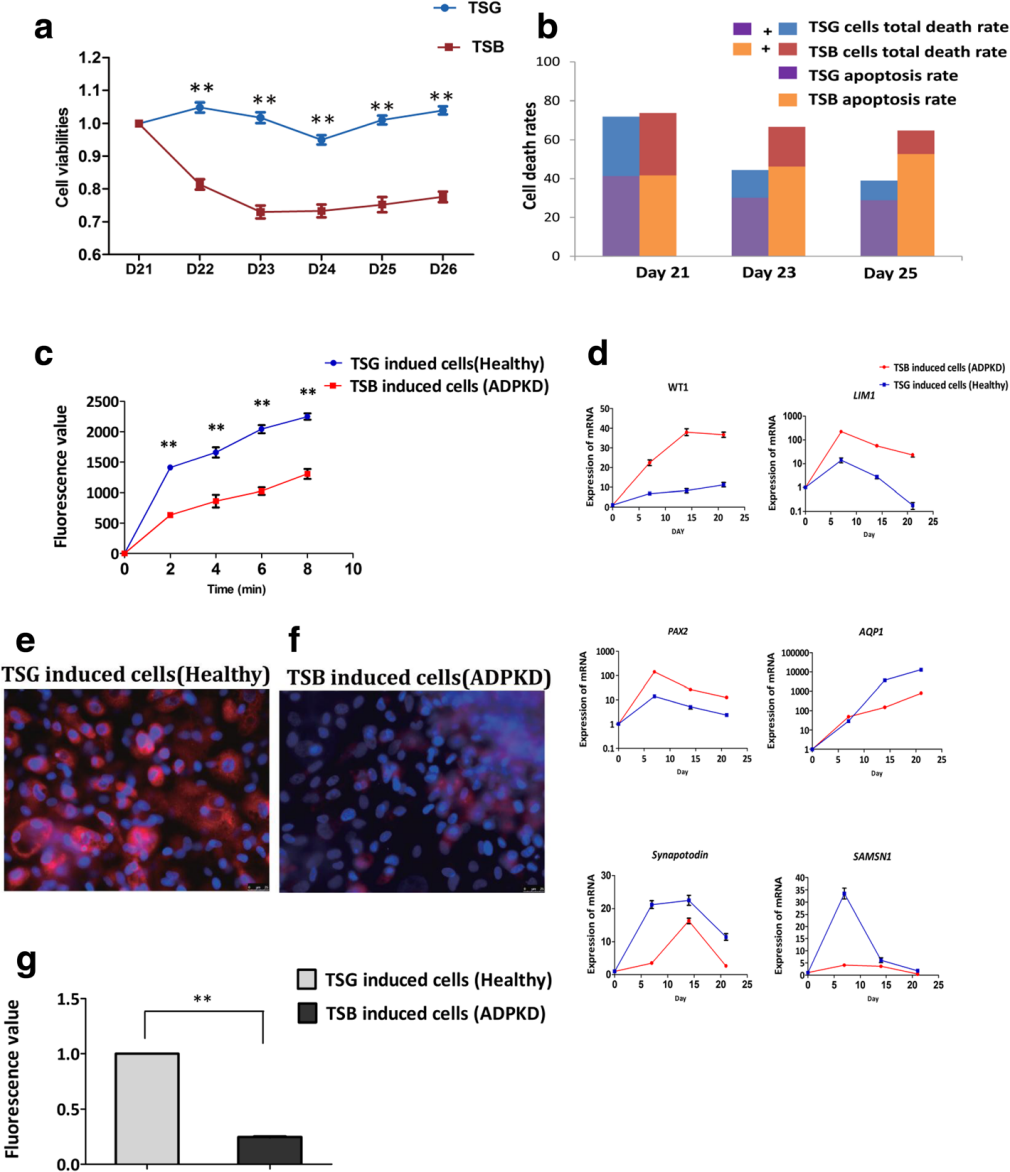
Differentiated iPSCs from an ADPKD patient and a healthy individual express different phenotypes. (**a**) Cell viabilities of TSG and TSB iPSCs tested by CCK-8 assays over the period from day 21 to day 28. Data presented as mean ± standard deviation from three independent sets of experiments, **P < 0.01. (**b**) Apoptosis rates of TSG and TSB iPSCs tested by Annexin V-FITC/PI staining over the period from day 21 to day 25. Data are averages of three independent experiments. (**c**) Water transportation assays carried out using induced TSG and TSB iPSCs. Data presented as mean ± standard deviation from three independent sets of experiments, **P < 0.01. (**d**) Marker genes of TSG and TSB iPSCs during the entire process of differentiating iPSCs to functional KLCs. Data presented as mean ± standard deviation from three independent sets of experiments. (**e**–**g**) BSA absorption assays of TSG and TSB KLCs derived from iPSCs. Bar = 25 μm. Data presented as mean ± standard deviation from three independent sets of experiments, **P < 0.01. ADPKD autosomal dominant polycystic kidney disease, TSB, TSG names of family members (Color figure online)

Because the ADPKD-iPSCs could be induced to differentiate into KLCs in the same way as the healthy human iPSCs or H9 ESCs, we investigated whether the phenotypes of the differentiated KLCs derived from ADPKD-iPSCs were different from those derived from healthy human iPSCs. During the induction process, no obvious differences in cell morphology were observed based on light microscopy (data not shown). However, the cell proliferation capacity was different by the CCK8 assay. The proliferation viabilities of ADPKD-iPSC differentiated cells dropped continuously compared to those of TSG iPSC differentiated cells at days 21–26 (Fig. 5a).

Previous studies have reported that increased apoptosis is an early event in ADPKD [28], therefore we analyzed the apoptosis rates of these two iPSC line differentiated cells by double labeling with Annexin V-FITC/propidium iodide (PI) followed by FCM. Early apoptotic cells were stained with Annexin V-FITC whereas late apoptotic cells and necrotic cells were stained with both Annexin V-FITC and PI. As shown in Fig. 5b, these two iPSC lines after differentiation for 21 days showed a similar percentage of cells undergoing early apoptosis (Annexin V-FITC staining only; 41.33% vs 41.71%). However, the percentage undergoing early apoptosis (Fig. 5b, orange bar) in ADPKD at day 23 was increased compared to the healthy cells (purple bar) (46.2% vs 30.12%), and the percentage undergoing late apoptosis in ADPKD at day 25 was significantly increased compared to the control cells (52.7% vs 28.88%). The results confirmed that the apoptosis rate of cells differentiated from ADPKD-iPSCs was higher than that from healthy iPSCs at days 21–25 of the induction.

Water transport ability is generally used to estimate kidney tubule function [29]. Using an improved version of the reported protocol [29], we measured the water transport ability of differentiated KLCs by measuring the fluorescent product of CFSE washed out under hypotonic conditions at 2-minute intervals. In this case, differentiated KLCs and HK2 cells both showed a similar response under hypotonic conditions (Additional file 1: Figure S1d). Surprisingly, the assay results also showed that the water transportation function of KLCs was much weaker in TSB iPSCs than in TSG iPSCs (Fig. 5c). HK2 cells were used as positive control. To estimate functional characteristics of KLCs, a cell permeability assay was used to determine the endocytic uptake of albumin as further evidence of glomerulus and podocyte-like functional characteristics [25]. After 28 days of differentiation, rhodamine-albumin was employed to detect the albumin absorption function of KLCs differentiated from these two iPSC lines. The intensity of red fluorescence decreased by 76% (*P* < 0.01) in TSB iPSC-KLCs compared to that of in TSG iPSC-KLCs (Fig. 5e–g).

To further investigate whether the expression level of *SAMSN1* changes in the process of kidney cell differentiation were consistent with the genomic changes between ADPKD patients and healthy individuals, we then performed qPCR for *SAMSN1* as well as other kidney-related genes (Fig. 5d). Expression levels were varied among the different genes but in general exhibited the same expression tendency in ADPKD-iPSCs and healthy iPSCs (Fig. 5d). *SAMSN1* mRNA levels in the ADPKD patient were lower than in the healthy individual almost throughout the induction period. Meanwhile the *AQP1* values were significantly lower in differentiated ADPKD-iPSCs than in healthy iPSCs (Fig. 5d), which might explain why comparable results were obtained in the water transport assay.

### Knockdown of *SAMSN1* may attenuate differentiation and/or function of KLCs in ADPKD

**Fig. 6:**
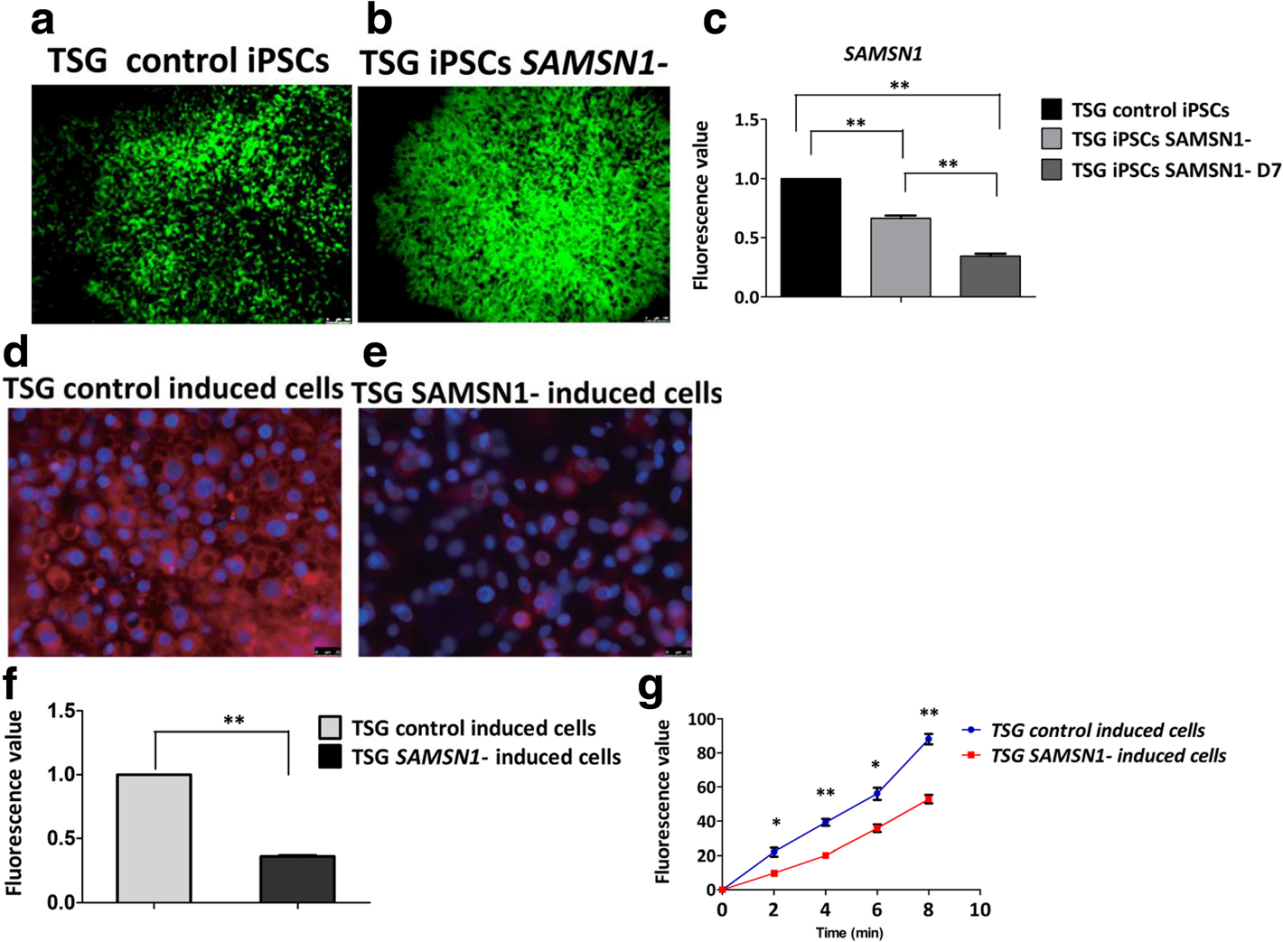
Knockdown of *SAMSN1* may attenuate differentiation and/or function of KLCs in ADPKD. (**a**–**c**) Morphology of TSG control induced cells and TSG *SAMSN1*-induced iPSCs and the relative expression rates of *SAMSN1* in TSG *SAMSN1*-induced iPSCs compared to those in TSG control-induced cells. Bar = 100 μm. Data presented as mean ± standard deviation from three independent sets of experiments, **P < 0.01. (**d**–**f**) BSA absorption assays of TSG control-induced cells and TSG *SAMSN1*-induced iPSCs. Bar = 25 μm. Data presented as mean ± standard deviation from three independent sets of experiments, **P < 0.01. (**g**) Results of water transportation assays of TSG control-induced cells and TSG *SAMSN1*-induced iPSCs. Data presented as mean ± standard deviation from three independent sets of experiments, *P < 0.05, **P < 0.01. ADPKD autosomal dominant polycystic kidney disease, iPSC induced pluripotent stem cell, TSG name of family member (Color figure online)

We hypothesized that deletion of the 5′ UTR of *SAMSN1* may reduce its expression and in turn attenuate differentiation or function of KLCs in ADPKD. TSG iPSCs infected with blank virus were used as control (Fig. 6a), and a stable TSG iPSC *SAMSN1* cell line infected with *shSAMSN1* virus was established using a lentivirus system (Fig. 6b). Using the qPCR assay, we found that the *SAMSN1* expression level was knocked down by 35% (*P* < 0.01) in TSG *SAMSN1* cells compared to control TSG iPSCs after puromycin selection for about 14 days (Fig. 6c). No apparent morphological differences were observed between them (data not shown). After kidney cell differentiation for 7 days, the *SAMSN1* expression level in TSG *SAMSN1* cells was decreased to below 40% (*P* < 0.01) compared to TSG control cells (Fig. 6c).

Next, we aimed to verify whether the biological behaviors of KLCs derived from TSG *SAMSN1* iPSCs was the same as that of ADPKD-iPSCs. Both TSG control iPSCs and TSG *SAMSN1* iPSCs underwent kidney differentiation at the same time. After 28 days of differentiation, rhodamine-albumin was also employed to analyze the albumin absorption function of these two induced iPSCs. The intensity of red fluorescence was decreased by 63% (*P* < 0.01) in TSG *SAMSN1-*induced iPSCs compared to that of in TSG control iPSCs (Fig. 6d–f). Moreover, the water transportation assay was also performed. TSG *SAMSN1-*induced iPSCs also had a slightly reduced water transport ability compared to those of TSG control cells (Fig. 6g). This indicates that the functionality of KLCs in the TSG *SAMSN1-*induced iPSCs was reduced compared to TSG control iPSCs. These two functional results were consistent with the earlier results (Fig. 5c, e–g). All of these results verified that some biological behaviors of KLCs derived from TSG *SAMSN1* iPSCs were changed like ADPKD compared to TSG control iPSCs. The deletion of the 5′ UTR of *SAMSN1* reduced its expression and may attenuate the differentiation or function of KLCs in ADPKD.

## Discussion

Although decades have passed since the discovery of *PKD1/PKD2* mutations in ADPKD, the pathogenesis of ADPKD remains unexplored and it remains unclear which other genes contribute to the pathogen of ADPKD. To personalize the study of the unique pathology of ADPKD, we first established and characterized ADPKD-iPSCs from a special ADPKD family without defects in the *PKD1/PKD2* genes. We also reported a novel method of inducing human iPSCs to differentiate into functional KLCs, and the differentiated KLCs derived from ADPKD or his healthy sibling had different phenotypes and functions. Further, we found a rare mutation in the 5′ UTR of the *SAMSN1* gene, which may attenuate KLCs differentiation or/and function in ADPKD.

The use of iPSCs for disease modeling is based on the fact that these cells are capable of self-renewal and can be differentiated into all types of cells of the human body, and can therefore be utilized for the preparation of different disease models to study disease pathogenesis. Moreover, disease-specific iPSCs could be of enormous use as far as development of specific therapeutic regimens/drugs is concerned. For example, this technique has been used to generate motor neurons from iPSCs of a patient with spinal muscular atrophy (SMA) that showed selective deficits compared to those derived from the child’s unaffected mother [6]. This was the first study to demonstrate that human iPSCs can be used to model the specific pathology in a genetically inherited disease. Subsequently, more and more reports have shown that iPSCs derived from specific diseases provide good models for disease [22, 30–32]. In the case of ADPKD, although pathogenesis was studied previously, pathogenesis remained undetermined mostly because of the pathogenic gene polymorphisms or the existence of a third pathogenic gene [16]. ADPKD-iPSCs have previously been generated successfully but the genotypes were rarely described [33] or involved *PKD* gene mutations [34, 35]. In our study, ADPKD-iPSC lines have been generated from a Chinese ADPKD family without *PKD1* or *PKD2* gene mutations. The special ADPKD-iPSCs are so different from other ADPKD-iPSCs reported previously that this could provide a new opportunity for the study of ADPKD pathogenesis.

There are no widely accepted protocols for directed differentiation of human iPSCs into kidney epithelial cells that give rise to kidney cysts; although ADPKD-iPSCs have been established and induced to differentiate into hepatoblasts/epithelial cells [34] and vascular cells [35], the differentiation capacity of kidney cells has not yet been investigated. In our study, we combined the currently existing protocols of differentiating stem cells into IM cells and established a new stepwise protocol for inducing ADPKD-iPSCs to differentiate into KLCs (Fig. 4a). From the morphological observations, we concluded that the differentiated KLCs contain two kinds of cell populations which were similar to human podocytes and HK2 cells (Fig. 4b). We found that expression levels of mesoderm gene *BRY* and kidney cell differentiation genes were significantly increased compared to corresponding ADPKD-iPSCs in these two populations of cells during kidney differentiation (Fig. 4c). Meanwhile, the expression levels of *NANOG* and *OCT4* were downregulated (Fig. 4d), and immunofluorescence and FCM analysis confirmed these results (Fig. 4d, e). In addition, the differentiated KLCs were positive for acetylated alpha-tubulin, marking the primary cilia protein from confocal image stacks (Additional file 1: Figure S1c). Three-dimensional projections of these stacks demonstrated primary cilia extending beyond the cell surface, as reported previously [35–37]. A recent study reported that differentiated cells from human ESCs displayed water transport functional characteristics of human primary renal proximal tubular cells [29]. We consulted and modified Narayanan et al.’s protocol [29]. SCFE, instead of calcein-AM, was employed to detect the cumulative fluorescence intensity using Synergy™ HT (Bio-Tek), and we found that differentiated KLCs and HK2 cells both showed a similar response tendency (Additional file 1: Figure S1d). Meanwhile, Song et al. [38] reported that the differentiated podocytes displayed podocyte-like functional characteristics by detecting the uptake of FITC-labeled albumin. Using this protocol, we also observed absorption of rhodamine-albumin in the cytoplasm of the differentiated cells (Fig. 5e, f). The primary podocytes were used as positive control cells (Additional file 1: Figure S1e). It was surprising to find that the ADPKD-iPSC (TSB) differentiated KLCs showed a downregulation in these two functional experiments compared to healthy controls (TSG) (Fig. 5f, g). For these experiments, we used KLCs differentiated from H9 ESCs, two ADPKD-iPSC clones (TSB and THB) and a normal iPSC clone (TSG). We present the results of patient TSB and healthy sibling TSG because these similar results have existed in two ADPKD-iPSCs. Taken together, these data indicate for the first time that ADPKD-iPSCs could be induced to differentiate into functional kidney cells but with lower functional characteristics compared to healthy control iPSCs.

As reported formerly, *SAMSN1* (also known as *HACS1/NASH1/SLY2*) is widely expressed in hematopoietic tissues, muscle, heart, brain, lung, pancreas, endothelial cells and myelomas, usually acts as an immunoinhibitory adaptor, and plays significant roles in the development and regulation of immunocytes such as B cells, T cells and mast cells in both mouse and human [39]. In our gene mutation study, we found that the ADPKD patients did not have any *PKD1* or *PKD2* gene mutations but did present a deletion in the 5′ UTR of the *SAMSN1* gene (Fig. 1c lower panel, d). Further, the *SAMSN1* gene expression levels of all ADPKD patients were lower than those of other healthy family persons (Fig. 1f). Besides this, a recent study revealed that *SAMSN1* induces *Rac1*-dependent membrane ruffle formation and regulates cell spreading and polarization by reorganizing the cytoskeletal actin network, which counteracts excessive B-cell spreading [40]. Podocytes also have polygonal epithelial morphology and large cytoplasmic extensions [27], which demand extensive remodeling of the actin network. In our case, the KLC populations have polarized polygonal epithelial morphology and large cytoplasmic extensions that might be induced or regulated by *SAMSN1*. Consequently we hypothesized that the defect in the 5′ UTR of the *SAMSN1* gene reduced the expression of *SAMSN1*, which in turn affected KLC differentiation or function in ADPKD. To test this we first knocked down the *SAMSN1* expression in TSG-iPSCs (TSG *SAMSN1* iPSCs) and then induced the TSG *SAMSN1* iPSCs to differentiate into KLCs, and found that *SAMSN1* expression was significantly decreased by approximately 70% at day 7 after KLC differentiation (Fig. 6c). Next, we tested the water transportation and albumin absorbing functions, and in these two assays these differentiated KLCs derived from TSG *SAMSN1* iPSCs both showed a downregulated tendency, which indicated that fewer functional KLCs and/or weaker functional KLCs were generated after *SAMSN1* knockdown. So we thought that *SAMSN1* may affect KLCs differentiation and/or function in ADPKD development. Taken together, our data suggest that the 5′ UTR deletion of *SAMSN1* may affect KLCs differentiation and/or function and provide a meaningful hint for the occurrence and development of ADPKD. Finally, there is a deficiency in this study that includes ten persons’ samples from one family. In order to further clarify the significance of *SAMSN1* mutation in ADPKD, we will continue to expand the sample size to verify the results of this article.

## Conclusions

These results revealed that special ADPKD-iPSCs without *PKD1/PKD2* gene mutations can be generated and induced to differentiate into functional KLCs using our modified differentiation protocol. We also show that the deletion mutation in *SAMSN1* might be involved in KLCs differentiation and/or function in ADPKD and thus provide a new perspective to illustrate the underlying mechanism in ADPKD. We believe that ADPKD-iPSC-KLCs hold huge potential to be used as versatile model systems for the study of kidney disease.

## Additional files

Additional file 1: Figure S1. The additional characterization analysis for ADPKD-iPSC and KLCs. (a): The timeline and culture conditions of induction of fibroblasts to iPSCs. Lower panel; phase contrast microscopy showing each of the three major steps. Bar = 100um. (b): AP staining for stemness of stem cells in iPSC lines. Bar = 100um. (c): Immunofluorescence photomicrographs showing primary cilia (arrow head) in KCLs were generated from iPSCs. Bar = 5um. (d): Water transportation assays were carried out between HK2 positive cells and KCLs. Data are represented as mean ± standard deviation from three independent sets of experiments. (e): The podocyte was used as a positive control and absorbed rhodamine-albumin. Bar = 25um. (JPG 1230 kb)

Additional file 2: Figure S2. The Sanger sequencing analysis for *PKD* in a Chinese ADPKD family. (a): The novel missense mutation c.17 G > A, *p.Arg6His* in *PKD2* was predicted by three program. (b): The list of all ten persons analyzed for the mutations. (c): The real sequencing pictures of all ten individuals in this family. (JPG 4280 kb)

Additional file 3: Figure S3. The comparative genomic hybridization (CGH) microarray analysis for *PKD* in a Chinese ADPKD family. (a): Representative image of CGH analyses of the *PKD1* and *PKD2* genes in patient TSB and healthy TSG. (b): qPCR verification of all eleven variants detected by CGH microarray in patient TSB and healthy TSG. Shown are the averages of three independent experiments. (JPG 3730 kb)

Additional file 4: Ethical approval file. (JPG 45 kb)

## Abbreviations

ADPKD: Autosomal dominant polycystic kidney disease
ALP/AP: Alkaline phosphatase
bFGF: Basic fibroblast growth factor
BMP7: Bone morphogenic protein 7
BSA: Bovine serum albumin
CCK8: Cell counting kit 8
CNV: Copy number variation
DAPI: 4,6-Diamino-2-phenyl indole
DMEM/F12: Dulbecco’s Modified Eagle’s medium/Nutrient Mixture F12
EBs: Embryoid bodies
EDTA: Ethylene diamine tetraacetic acid
EGFP: Enhanced green fluorescent protein
ESC: Embryonic stem cell
ESRD: End-stage renal disease
FBS: Fetal bovine serum
FCM: Flow cytometry
HK2: Human kidney 2
hVEGF: Human vascular endothelial growth factor
IM: Intermediate mesoderm
iPSC: Induced pluripotent stem cell
KLC: Kidney-like cell
KSR: Knockout serum replacement
LR-PCR: Long-range PCR
NEAA: Nonessential amino acids
PFA: Paraformaldehyde
PI: Propidium iodide
*PKD1*: Polycystin-1
*PKD2*: Polycystin-2
RA: Retinoic acid
REGM: Renal epithelium growth medium
SMA: Spinal muscular atrophy
STR: Short tandem repeat
TBST: Tris-buffered saline containing Tween-20
Vc: Vitamin C
VPA: Valproic acid
β-ME: β-Mercaptoethanol
