# Supplementary figures and images for: Generation of special autosomal dominant polycystic kidney disease iPSCs with the capability of functional kidney-like cell differentiation
Source: Stem Cell Res Ther. 2017 Sep 19;8:196. doi: 10.1186/s13287-017-0645-8 (PMC5606115; doi:10.1186/s13287-017-0645-8)

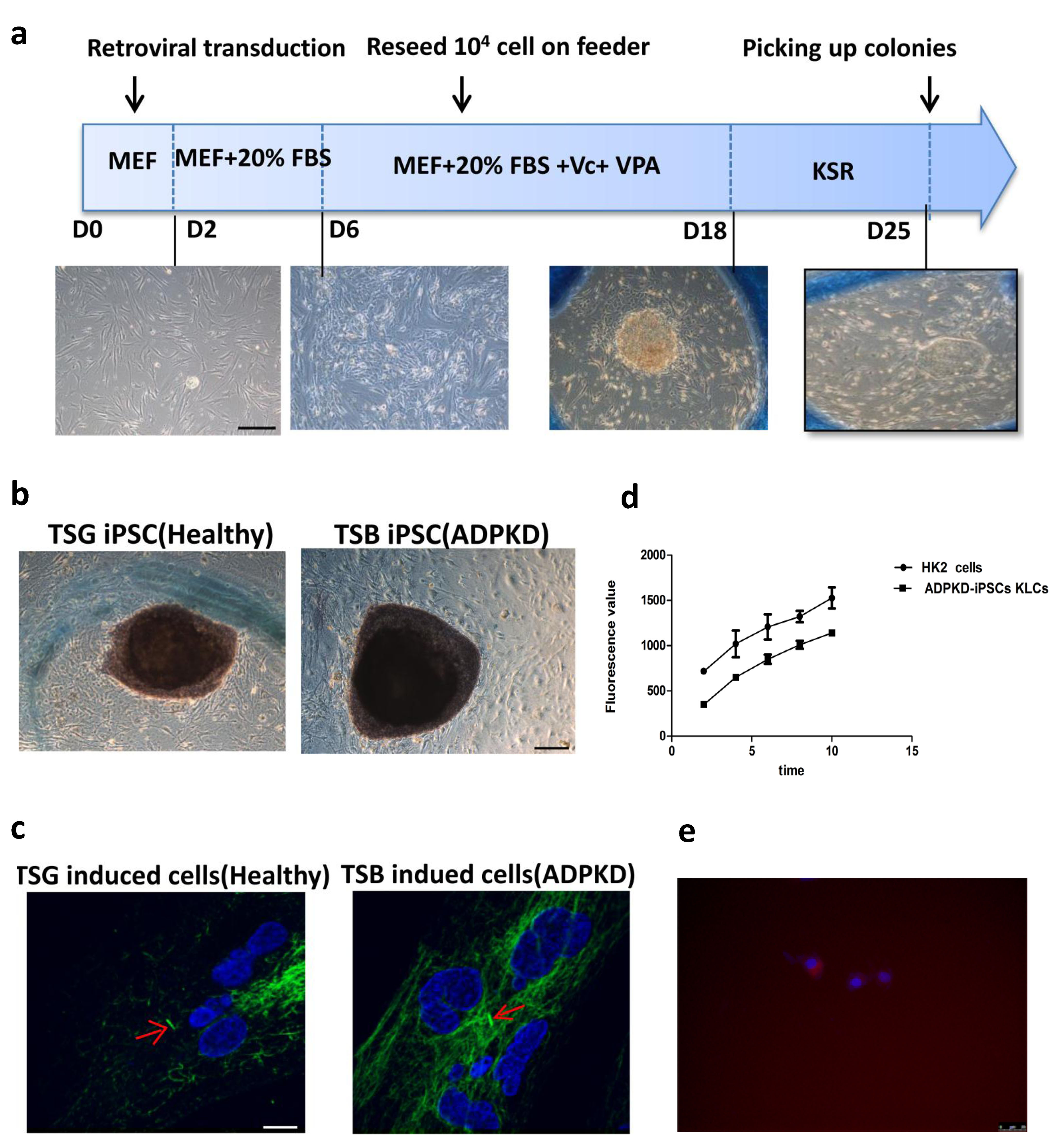

Supplement: Supplementary file 1 — The additional characterization analysis for ADPKD-iPSC and KLCs. (a): The timeline and culture conditions of induction of fibroblasts to iPSCs. Lower panel; phase contrast microscopy showing each of the three major steps. Bar = 100um. (b): AP staining for stemness of stem cells in iPSC lines. Bar = 100um. (c): Immunofluorescence photomicrographs showing primary cilia (arrow head) in KCLs were generated from iPSCs. Bar = 5um. (d): Water transportation assays were carried out between HK2 positive cells and KCLs. Data are represented as mean ± standard deviation from three independent sets of experiments. (e): The podocyte was used as a positive control and absorbed rhodamine-albumin. Bar = 25um. (JPG 1230 kb) [file 13287_2017_645_MOESM1_ESM.jpg]

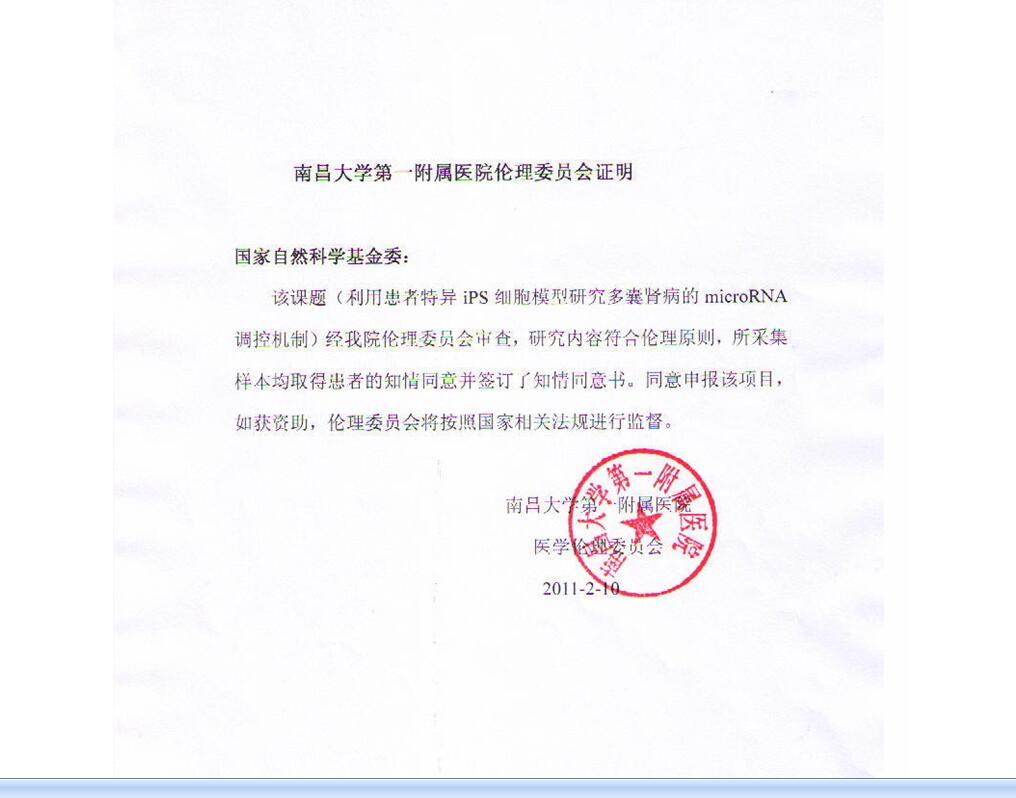

Supplement: Supplementary file 4 — Ethical approval file. (JPG 45 kb) [file 13287_2017_645_MOESM4_ESM.jpg]
